# Discovery of novel ATAD2 bromodomain inhibitors that trigger apoptosis and autophagy in breast cells by structure-based virtual screening

**DOI:** 10.1080/14756366.2020.1740924

**Published:** 2020-03-16

**Authors:** Dahong Yao, Jin Zhang, Jinhui Wang, Dabo Pan, Zhendan He

**Affiliations:** a Guangdong Key Laboratory for Genome Stability & Human Disease Prevention, School of Pharmaceutical Sciences, Shenzhen University, Shenzhen, China; b Shenzhen Key Laboratory of Novel Natural Health Care Products, Innovation Platform for Natural small molecule Drugs, Engineering Laboratory of Shenzhen Natural small molecule Innovative Drugs, Shenzhen University Health Science Center, Shenzhen, China; c State Key Laboratory of Biotherapy and Cancer Center, West China Hospital, Sichuan University, Collaborative Innovation Center for Biotherapy, Chengdu, China; d Shenzhen Honghui Bio-Pharmaceutical Co. Ltd., Shenzhen, China; e Institute of Traditional Chinese Medicine & Natural Products, Guangdong Province Key Laboratory of Pharmacodynamic Constituents of TCM and New Drugs Research, College of Pharmacy, Jinan University, Guangzhou, P. R. China

**Keywords:** ATAD2, breast cancer, Virtual Screening, apoptosis, autophagy

## Abstract

ATAD2 has been reported to play an important role in the processes of numerous cancers and validated to be a potential therapeutic target. This work is to discover potent ATAD2 inhibitors and elucidate the underlying mechanisms in breast cancer. A novel ATAD2 bromodomain inhibitor (AM879) was discovered by combining structure-based virtual screening with biochemical analyses. AM879 presents potent inhibitory activity towards ATAD2 bromodomain (IC_50_ = 3565 nM), presenting no inhibitory activity against BRD2-4. Moreover, AM879 inhibited MDA-MB-231 cells proliferation with IC_50_ value of 2.43 µM, suppressed the expression of c-Myc, and induced significant apoptosis. Additionally, AM978 could induce autophagy via PI3K-AKT-mTOR signalling in MDA-MB-231 cells. This study demonstrates the development of potent ATAD2 inhibitors with novel scaffolds for breast cancer therapy.

## 1. Introduction

**Figure 1.**
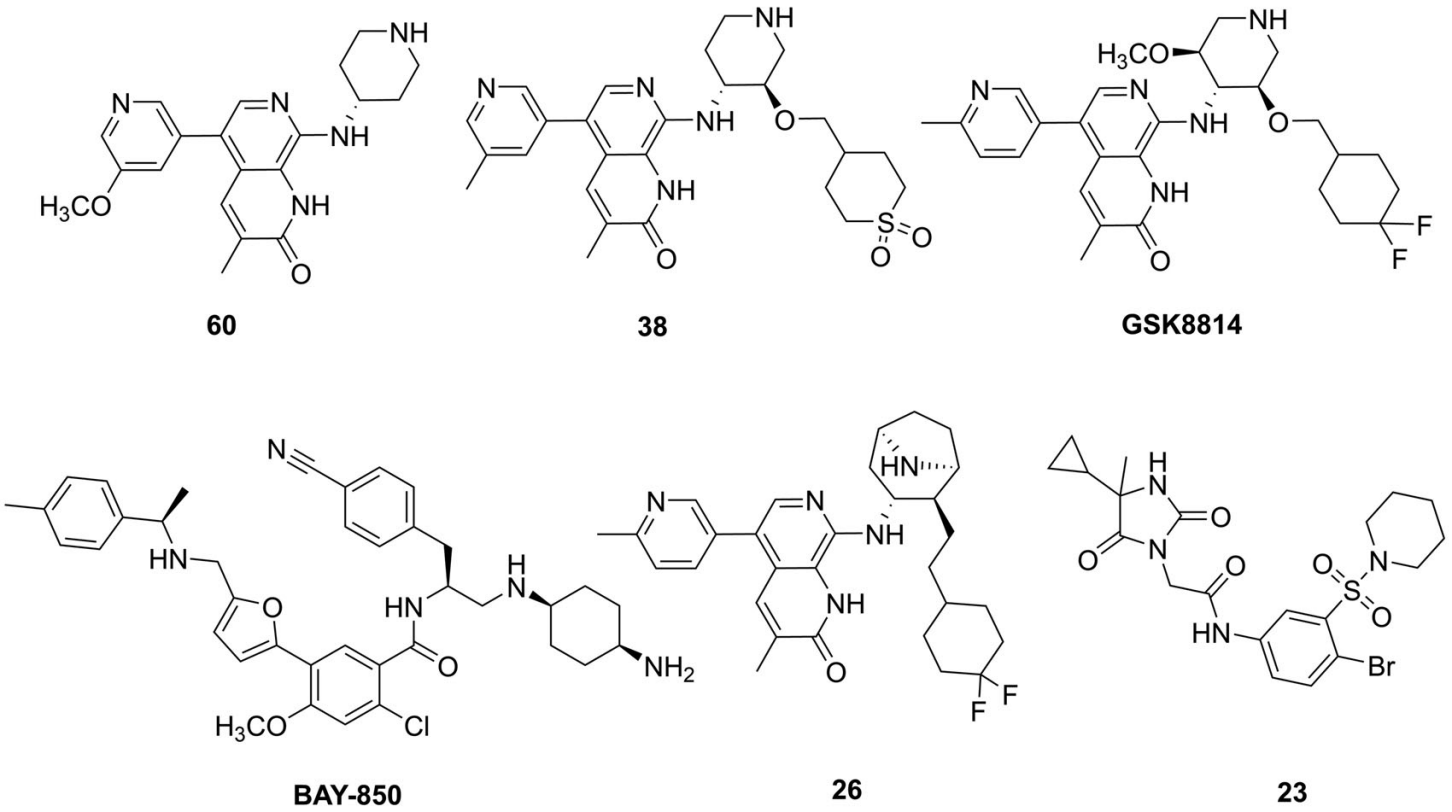
Structures of several ATAD2 inhibitors.

Histone acetylation modification is one of the important epigenetic regulatory methods, which functions as a regulator in numerous biological processes, including transcription, cell proliferation, differentiation, apoptosis and signal transduction^1^^,^^2^. The abnormality of histone lysine acetylation level is closely related to the occurrence of various diseases, especially in inflammation and tumour^3^. Bromodomain-containing proteins, readers of histone acetylation, function to regulate transcription programmes associated with phenotypic changes^4^. It is validated that disrupting the protein-protein interactions between BET protein and Histone acetylated lysine has emerged as a promising target for various cancers^5^. ATAD2, also known as ATAD2A or ANCCA (AAA nuclear coregulator cancer-associated protein), belongs to the bromodomain and extra-terminal family domain (BET) IV family, could recognise acetylated lysine residues at K5 and K12 on histone H4^6^. ATAD2, as a nuclear protein, mainly expresses in embryonic stem and germ cells which plays an essential role in chromatin remodelling^7^. Accumulating studies have demonstrated that ATAD2 is highly expressed in numerous cancers, including lung adenocarcinoma, colorectal, gastric and breast cancer^8–11^. ATAD2 is a valid and promising therapeutic target involved in cell survival, proliferation, apoptosis, and migration for several types of human cancers^12–14^. Mechanistically, ATAD2 can regulate multiple oncogenes, such as *c-Μyc*, *E2F1* and *cyclin D1*, which are capable of contributing to cancer cells survival via enhancing cell proliferation, invasion and migration^15^. ATAD2 positively regulates the oncogene AIB1/ACTR expression, and serves as a transcriptional co-regulator for AR and ERα, resulting in elevating the expression of genes that promote cancer cell proliferation and survival^16^. Additionally, ATAD2 could positively mediate the expression of pro-survival genes, including SGK, VEGF, IRS2 and Akt, which contribute to promoting breast cancer progression^17^. Structurally, ATAD2 possesses a bromodomain and an ATPase domain. The bromodomain (BRD) is composed of conserved left-handed four-helix bundle structure, and the ZA and BC loops are responsible for identifying and binding to acetylated lysine residues at K5 and K12 on histone H4^18^. The ATAD2 bromodomain has attracted accumulating efforts for drug discovery for oncology. Recently, several potent and selective ATAD2 inhibitors have been reported (Figure 1). Three hits binding to ATAD2 were discovered based on a fragment-based screen strategy by NMR and X-ray crystal technology in 2014, which validated ATAD2 bromodomain should be drugable^19^. The first known low-micromolar inhibitor (**60**) of the ATAD2 bromodomain was designed based on a fragment-based method with IC_50_ value of 1.3 µM, but showing a similar inhibition for BRD4^20^. Paul Bamborough et al. reported a selective ATAD2 inhibitor (**38**) through structure-based optimisation of a series of naphthyridones which bears 100 nM ATAD2 potency and 100-fold BET selectivity^21^. Additionally, a chemical probe (**GSK8814**) for the ATAD2 bromodomain was reported with a high affinity and selectivity for ATAD2. Disappointingly, it presented weak antiproliferative *in vivo*^22^. Additional several small molecule inhibitors were developed, such as **BAY-850**^23^, **26**^24^ and **23**^25^, despite their high affinity for ATAD2, they lack activity *in vitro* or *vivo* cellular assays. Therefore, it is very urgent to develop new inhibitors targeting ATAD2 that exerts high affinity, selectivity and potent antiproliferatory activity for cancer cells *in vitro* and *vivo.*

In this study, we discovered a novel inhibitor (AM879) of ATAD2 employing the structure-based virtual screening approach, which exhibits moderate inhibitory activity against ATAD2 with IC_50_ value of 3,565 nM against ATAD2, presenting no inhibitory activity against BRD2-4. AM879 exhibited potent antiproliferative activity in a dose- and time-dependent manner (IC_50_ = 2.43 µM, 24 h). Additionally, we proposed the binding mode of AM879 and ATAD2 by molecular docking and dynamics simulation. *In vitro*, AM879 could significantly downregulate the expression of c-Myc, and induced obvious apoptosis in MDA-MB-231 cells. Intriguingly, we found that AM978 induced autophagy via PI3K-AKT-mTOR signalling. Collectively, these findings shed light on a novel and promising lead compound for the development of ATAD2 inhibitors and characterised the biological function for breast cancer therapy.

## 2. Methods

### 2.1. Molecular docking

Discovery Studio 3.5 was employed by docking the ligands to ATAD2. The structure of ATAD2 (PDB code: 5LJ0) was extracted from PDB data bank (https://www.rcsb.org). The important crystal water was retained for keeping the stability of the crystalline water network in Tyr1021, WAT and ligand^22^. The ligand library containing over 1,700,000 compounds from Specs and Chemdiv databases was prepared and filtered for Lipinski’s rule of five and veber rules. The top 30% compounds based on the docking scores were used to redock to ATAD2 using CDOCKER module, Furthermore, to ensure the rationality of the binding mode and the structural diversities of compounds, 200 candidates were selected.

### 2.2. Molecular dynamics (MD) simulations

The MD simulation was performed by Amber 10 package^26^. The first restraining energy minimisation was carried out by the steepest descent method with 0.1 kcal/mol Å2 restraints for all atoms of the complexes for 5000 steps. And then, we removed the restraints of ligand (only restraining the protein) to perform the second energy minimisation, and another energy minimisation was made under releasing all the restraints. 5000 steps were set for each energy minimisation. To handle the long-range Coulombic interactions, the particle mesh Ewald (PME) summation was used. The SHAKE algorithm was employed on all atoms covalently bonded to a hydrogen atom, allowing for an integration time step of 2 fs in the equilibration and subsequent production runs. The annealed programme was from 0 to 310 K for 50 ps. Under releasing all the restraints, the system was again equilibrated for 500 ps. The production phase of the simulations was run without any restraints for a total of 100 ns.

### 2.3. Tr-FRET assay

The assay was performed by TR-FRET technology using recombinant bromodomain and BET Ligand^27^. The compounds were diluted in 100% DMSO. They were diluted 20 fold in 5% DMSO in Reaction Buffer and 2 µl of the dilution was added to a 20 µl reaction. The reaction mixture incubated for 180 min prior to reading the TR-FRET signal. TR-FRET was recorded as the ratio of the fluorescence of the acceptor and the donor dyes (acceptor/donor). Binding experiments were performed in duplicate at each concentration. The TR-FRET data were analysed using the computer software, Graphpad Prism.

### 2.4. Cell culture, antibodies and reagents

MDA-MB-231cells were purchased from American Type Culture Collection (ATCC, Manassas, VA, USA). Cells were cultured in DMEM with 10% foetal bovine serum and incubated with 5% CO2. Antibodies used in this study were as follow: ATAD2 (ab176319, abcam), c-Myc (ab32072, abcam), cMyc^Ser62^ (ab51156, abcam), Bax(5023, CST), Bcl-2(15071, CST), caspase8(9746, CST), caspase-3 (9665, CST), AKT (4691, CST), mTOR (2983, CST), p-mTOR^Ser2448^ (5536,CST), ULK1 (6439, CST), p-ULK1^Ser555^ (5869, CST), p62 (5114, CST), Beclin1 (4122, CST), LC3B (3868, CST), β-actin (3700, CST).

### 2.5. Cell viability assay

Cells were dispensed in 96-well plates at a density of 5 × 104 cells/ml. After 24 h incubation, cells were treated with different concentrations of compounds for the indicated periods. Cell viability was measured by the MTT assay.

### 2.6. Autophagy and apoptosis assays

For the autophagy assay, MDA-MB-231 cells were transfected with GFP/mRFP-LC3 then treated with 2.5 µM AM879 for 24 h and the autophagy ratios were observed under a fluorescence microscope. For apoptosis assay, MDA-MB-231 cells were treated with 2.5 µM AM879 and apoptosis ratios were determined by flow cytometry analysis of Annexin-V/PI double staining. TUNEL staining was also used to detect cell apoptosis. Cells were treated with DMSO or AM879 and then formalin-fixed. TUNEL staining was performed using a One-Step TUNEL Apoptosis Assay Kit (Beyotime, C1086) according to the manufacturer’s instructions, with apoptotic cells exhibiting green nuclear fluorescence.

### 2.7. Immunofluorescence analysis

The MDA-MB-231 were sequentially incubated, starting with ATAD2 antibody (1:200) and p-cMyc antibody (1:200) diluted in PBS containing 1% BSA incubated overnight at 4 °C, followed by addition of fluorescent-labeled secondary antibodies (TRITC, ab6718; Alexa Fluor 488, ab150077) for 1 h at room temperature.

### 2.8. Western blot

Adherent and floating cells were collected after treatment with AM879 for indicated times. Cells were lysed in a lysis buffer consisting of Hepes 50 mM pH 7.4, Triton-X-100 1%, sodium orthovanada 2 mM, sodium fluoride 100 mM, edetic acid 1 mM, PMSF 1 mM, aprotinin (Sigma, MO, USA) 10 mg/L and leupeptin (Sigma) 10 mg/L at 4 °C for 1 h. After 12,000 rpm centrifugation for 15 min, the protein content of supernatant was determined by the Bio-Rad DC protein assay (Bio-Rad Laboratories, Hercules, CA, USA). Equal amounts of the total protein were separated by 10–15% SDS-PAGE and transferred to PVDF membranes, and the membranes were soaked in blocking buffer (5% skimmed milk or BSA). Proteins were detected using primary antibodies, followed by HRP-conjugated secondary antibody and visualised by using ECL as the HRP substrate. Quantity One 4.4 was used to quantify.

### 2.9. Statistical analysis

The results were expressed as means ± SEM. Differences between two groups were processed by Student’s unpaired *t*-test by GraphPad Prism 7.00 software. Multi-groups comparisons of the means were carried out by one-way analysis of variance (ANOVA) test with *post hoc* contrasts by the Student–Newman–Keuls test. All the presented data was confirmed by at least three independent experiments. *p* < 0.05 was considered statistically significant.

## 3. Results and discussion

### 3.1. The discovery of potential hits by virtual screening

**Figure 2.**
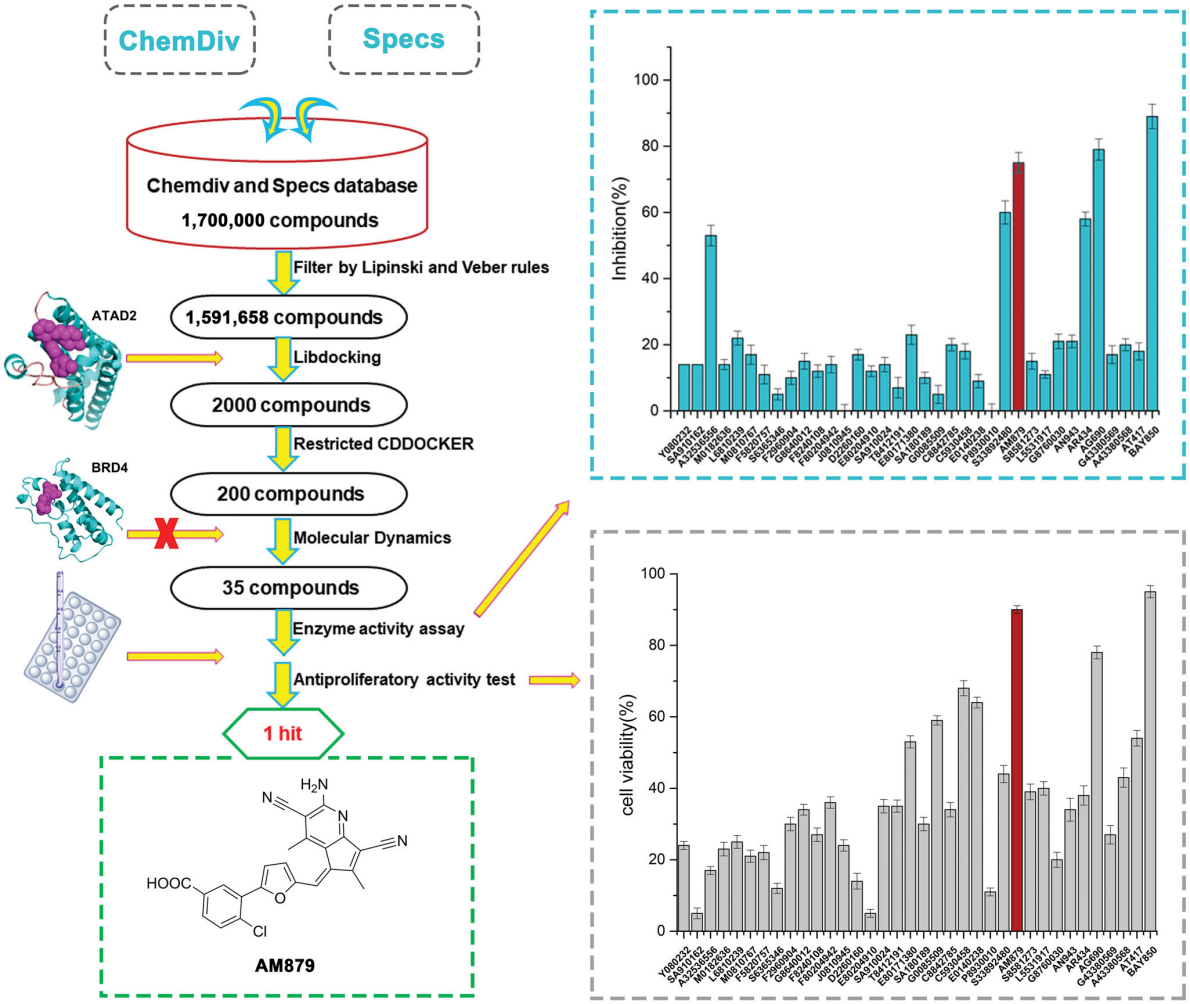
Virtual screening schematic model for the discovery process of novel ATAD2 inhibitors.

In order to discover potential ATAD2 inhibitors with novel skeleton, we adopt a comprehensive screening strategy based on virtual screening and biochemical analyses (Figure 2(A)). Firstly, in order to eliminate compounds with unfavourable drug-like descriptors and physicochemical properties, the Chemdiv and Specs chemical library containing 1,700,000 compounds were filtered by Lipinski’s rule of five and veber rules to yield 1,591,658 compounds. Subsequently, these compounds were applied in structure-based pharmacophore (SBP) virtual screening through the Libdocking protocol in Discovery Studio 3.5 (DS). Only compounds ranked 2000 according to the fit scores were retained to access to next docking. Top 200 hits bearing better -CDOCKER interaction energy were further screened by CDOCKER protocol. To characterise the selectivity of these hits over BRDS, we further remove these compounds binding well with BRD4 through molecule docking. Additionally, molecular dynamics based on AMBER 10 were performed to further screen to product 35 hits carrying diverse scaffolds. Subsequently, we conducted the ATAD2 inhibitory activity assay using TR-FRET technology to investigate the potency of 35 hits purchased commercially (Table 1). The results showed that there are five hits exerting higher than 50% inhibition rate at 20 µM (Figure 2(B)). Among them, AM879 and AG690 presented favourable activity with enzyme inhibition rate 75% and 79% respectively, BAY850 was used as the positive control drug. In cell viability assay, AM879 showed a potent antiproliferatory activity with 90% inhibition rate at 20 µM, while AG690 presented less potency than AM879. Collectively, the findings suggested that AM879 is a potential ATAD2 bromodomain inhibitor with cell antiproliferatory activity in MDA-MB-231 cells.

**Table 1.** The chemical structures of selected candidates.

Compd.	Compd. ID	Structure	Docking score
1	Y080232		−8.07
2	SA910162		−7.97
3	A32536556		−2.81
4	M0182636		−5.40
5	L6810239		−7.05
6	M0810767		−7.04
7	F5820757		−7.01
8	S6365346		−7.01
9	F2960904		−6.85
10	G8640012		−7.57
11	F8240108		−7.29
12	F80204942		−9.21
13	J0810945		−7.33
14	D2260160		−7.18
15	E80204910		−7.84
16	SA910024		−8.07
17	T8412191		−7.51
18	E80171380		−7.03
19	SA180189		−6.99
20	G0085509		−6.94
21	C8842785		−6.86
22	C5930458		−7.07
23	E0140238		−6.96
24	P8930010		−6.99
25	S33892480		−7.85
26	AM879		−11.32
27	S8581273		−7.07
28	L5531917		−6.85
29	G8760030		−7.13
30	AN-943		−7.08
31	AR434		−7.44
32	AG-690		−8.29
33	G43380569		−8.17
34	A43380568		−7.20
35	AT-417		−6.90

### 3.2. Docking and molecular dynamic (MD) simulation of AM879 with ATAD2

**Figure 3.**
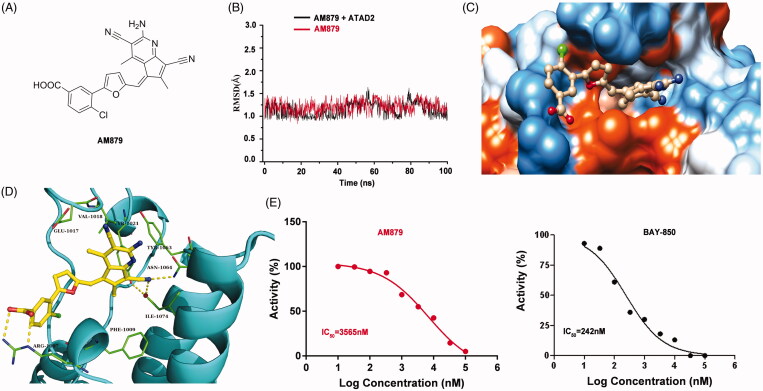
AM879 is a novel potent ATAD2 Bromodomain inhibitor. (B) The time evolution of RMSD of backbone atoms for the residues around 5 Å of AM-879 and heavy atoms of AM879 (C) The detailed interaction between AM-879 and ATAD2. (D) The polar (sky blue) and nonpolar (yellow orange) interaction surface between AM879 and ATAD2. (E) Inhibitory effect of AM879 on ATAD2 Bromodomain.

To explore the interaction mode of AM-879 against ATAD2, a computational study including molecular docking, molecular dynamics simulation and binding free energy calculation were performed. The root-mean-square deviation (RMSD) of the heavy ligand atoms and the backbone atom of protein around 8 Å ligand were assessed in 100 ns MD simulation, the RMSD fluctuated between 0.75 and 1.5 indicated the system was a well-behaved setup (Figure 3(A)). The binding free energy between AM879 and ATAD2 was −26.55 kcal/mol, the nonpolar term (−35.79 kcal/mol) played a primary role in AM-879 binding to ATAD2 (Table 2). Furthermore, a detailed view of the interaction of compound AM-879 with ATAD2 was displayed in Figure 3(B,C). As presented, the 5H-cyclopenta [b] pyridine group of AM-879 penetrate deeply into the hydrophilic pocket composed of the residues Tyr1021, Tyr1063, Asn1064 and ILE1074 by the polar interactions. A conserved hydrogen bond was monitored between Asn1064 and cyanogroup of AM879. A water molecule hydrogen bond network was initiated by cyanogroup and Tyr1021, which contributed to stabilising the ligand through additional hydrogen bonds. Interesting, the middle tetrahydrofuran linker section formed strong nonpolar interaction with the hydrophobic pocket. In addition, the Carboxylic acid of AM879 could act as a hydrogen bond receptor, initiating two direct hydrogen bonds with the conserved arginine (Arg1007) to contribute to stabilising the ligand. These results indicated that compound AM879 could bind to ATAD2 stably. Next, we conducted the Time-Resolved Fluorescence Resonance Energy Transfer (TR-FRET) Assay to confirm the inhibitory activity of AM879 against ATAD2 bromodomain. The results revealed that AM879 showed potent inhibitory activity against ATAD2 bromodomain with IC_50_ of 3565 nM and BAY-850 was used as the reference compound (IC_50_ =242 nM) (Figure 3(E)). To further investigate the selectivity of AM-879, the binding affinities of AM-879 were measured by TR-FRET against the first and second bromodomains of BRD2, BRD3 and BRD4 (Table 3). The results revealed that AM-879 showed no activity against BRD2, BRD3 and BRD4. Collectively, these findings substantiated that AM879 is a novel potent selective ATAD2 bromodomain inhibitor.

**Table 2.** The binding free energies were calculated by MM-GBSA method (kcal/mol)

Compd.	ΔE_ele_	ΔE_vdw_	ΔE_MM_	ΔG_sol-np_	ΔG_sol-ele_	ΔG_sol_	ΔG_polar_^a^	ΔG_nonpolar_^b^	ΔG_bind_
AM879	−64.11	−31.34	−95.45	−4.46	73.36	68.90	9.25	−35.79	−26.55

^a^ΔG_polar_ = ΔE_ele_ +ΔG_sol-ele_; ^b^ΔG_nonpolar_ =ΔE_vdw_ +ΔG_sol-np_.

**Table 3.** Binding affinities of AM-879 were measured by TR-FRET against ATAD2 and the first and second bromodomains of BRD2, BRD3 and BRD4.

	IC_50_ (μM)
ATAD2	3.56
BRD2(1)	>50
BRD2(2)	>50
BRD3(1)	>50
BRD3(2)	>50
BRD4(1)	>50
BRD4(2)	>50

### 3.3. Am879 inhibits ATAD2 activity in MDA-MB-231 cells

**Figure 4.**
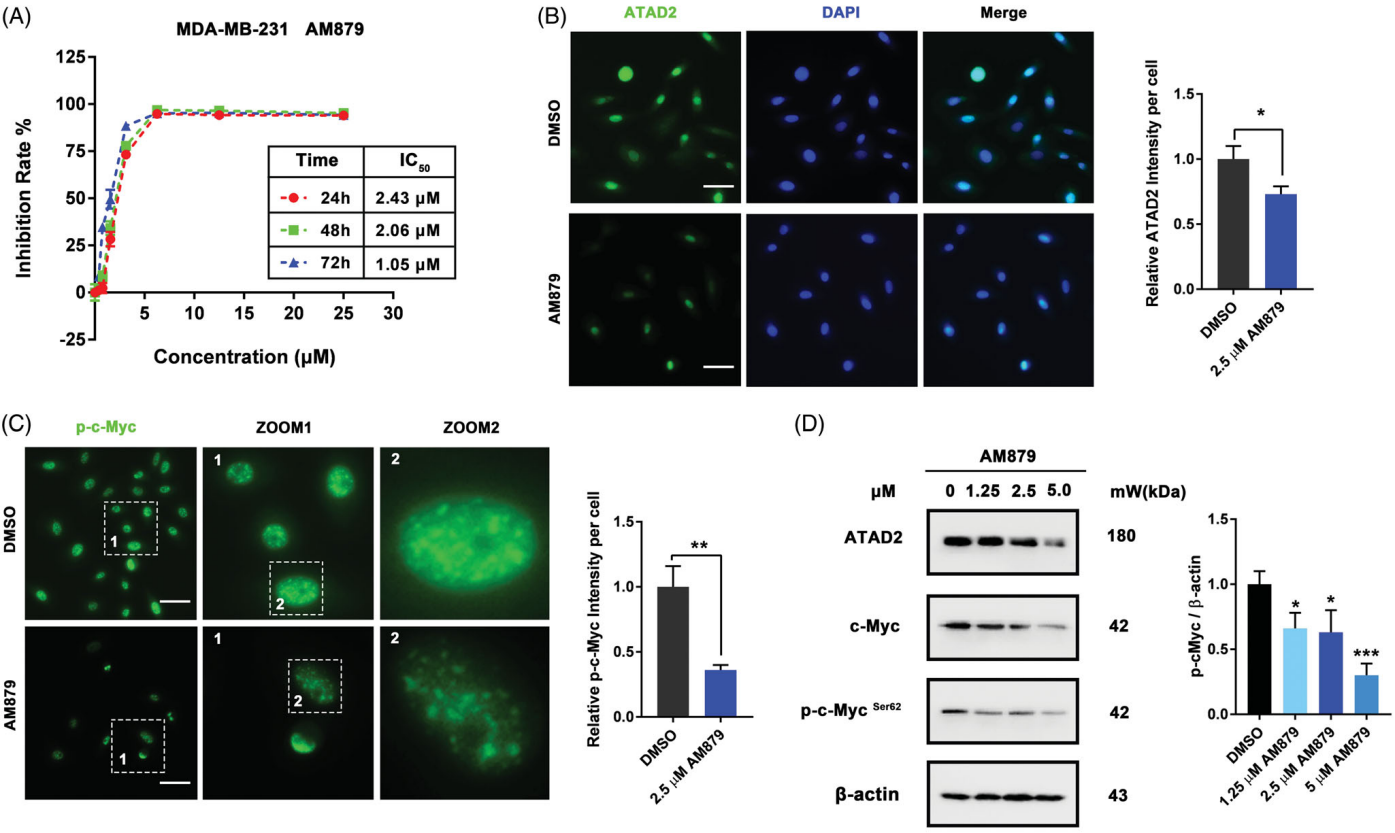
AM879 inhibits ATAD2 activity in MDA-MB-231 cells. (A) MTT assays were performed to measure the antiproliferative potency of AM879 against MDA-MB-231 cells. (B) MDA-MB-231 cells were treated with 2.5 µM AM879 then the expression of ATAD2 was detected by immunocytochemistry. Green: anti-ATAD2; Blue: DAPI. Scale bar = 50 µm. (C) MDA-MB-231 cells were treated with 2.5 µM AM879 then the expression of p-cMyc was detected by immunocytochemistry. Green: anti-p-cMyc. Scale bar = 50 µm. (D) Cells were treated with 1.25, 2.5 and 5.0 µM AT879 for 24 h, then the expressions of ATAD2, c-Myc and p-cMyc were detected by western blot analysis. β-actin was measured as the loading control.

To further evaluate the *in vitro* activity of AM879, we firstly detected the anti-proliferation activity of AM879 on MDA-MB-231, MDA-MB-436, MDA-MB-468, BT474 and MCF-7 cells (Figure S1). AM879 showed potent antiproliferatory activity against these cancer cell. Especially, AM879 exhibited potent antiproliferative activity in a dose- and time-dependent manner (IC_50_ = 2.43 µM, 24h; IC_50_ = 2.06 µM, 48 h; IC_50_ = 1.05 µM, 72 h) in MDA-MB-231 cells (Figure 4(A)). Next, we evaluated the effect of AM978 on the expression of ATAD2 and the ATAD2 intensity was weak after AM879 treatment (Figure 4(B)). In addition, the phosphorylation of ATAD2 downstream substrate c-Myc was also decreased which further confirmed the inhibition effect of AM879 on ATAD2 (Figure 4(C)). Furthermore, the western blot results also demonstrated that AM879 inhibited the expression of ATAD2, c-Myc and the phosphorylation of c-Myc (Figure 4(D)). These results indicated that AM879 functioned as an ATAD2 inhibitor in MDA-MB-231 cells.

### 3.4. Am879 induced apoptosis in MDA-MB-231 cells

**Figure 5.**
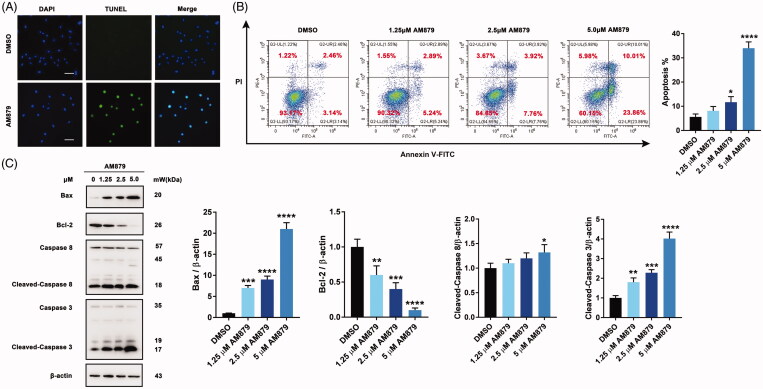
AM879 induced apoptosis in breast cancer cells. (A) MDA-MB-231 cells were treated with 2.5 μM AM879 for 24 h, apoptosis was evaluated by TUNEL assay. Scale bar= 40 μm. (B) MDA-MB-231 cells were treated with 1.25, 2.5 and 5 μM AM879 for 24 h, apoptosis ratios were determined by flow cytometry analysis of Annexin-V/PI double staining. (C)Western blot analysis of Bax, Bcl-2, Caspase8 and Caspase3 in MDA-MB-231 cells treated with 1.25, 2.5 and 5 μM AM879 for 24 h. Relative Bax, Bcl-2, Caspase8 and Caspase3 expression levels were quantified by normalisation to β-actin. ns, not significance; *p < 0.05, **p < 0.01, ***p < 0.001 and ****p < 0.0001, compared to DMSO treated Control.

Considering the close relationship between c-Myc and apoptosis, we next checked whether apoptosis was involved in the antiproliferative mechanism of AM879. Firstly, TUNEL assay was performed to examine whether AM879 could induce apoptosis and obvious FITC fluorescence were aggregated in the nucleus after AM879 treatment (Figure 5(A)). Next, Annexin-V/PI staining analysis revealed that a significant increase in early and late apoptotic cells in the presence of AM879 was observed, indicating that AM879 could elicit obvious apoptosis (Figure 5(B)). Additionally, AM879 also substantially elevated the expression of Bax, reduced the expression of Bcl-2, accompanied with the cleavage of caspase3 and caspase8, which suggested the activation of the apoptosis (Figure 5(C)). Therefore, AM879 is capable of inducing apoptosis in MDA-MB-231 breast cancer cells.

### 3.5. Am978 induced autophagy via PI3K-AKT-mTOR inhibition in MDA-MB-231 cells

**Figure 6.**
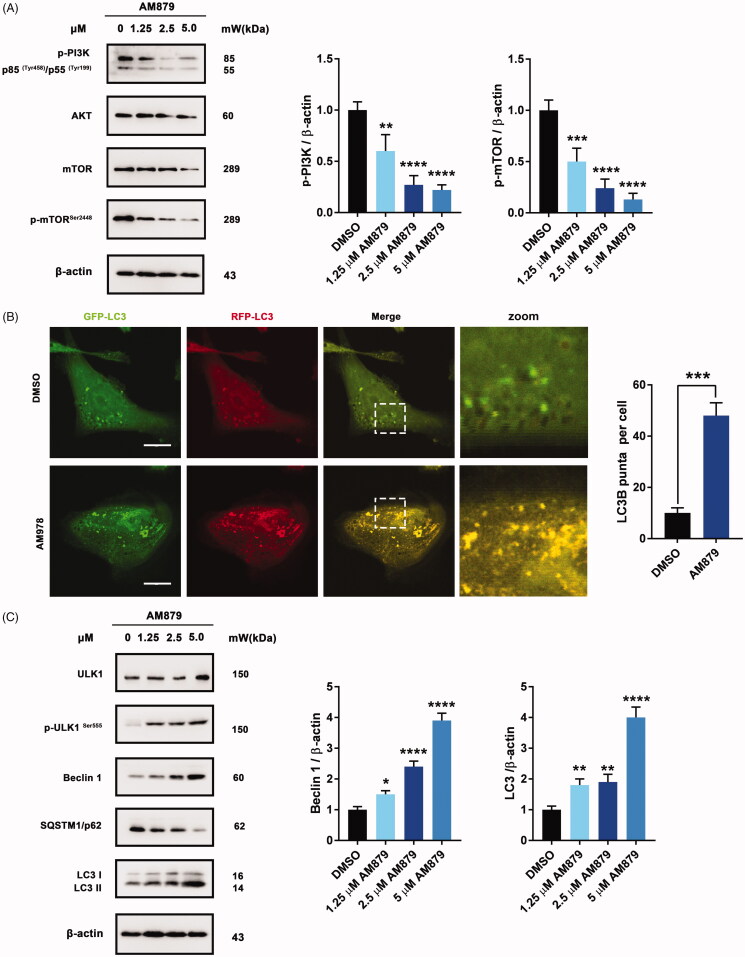
AM879 induced autophagy in MDA-MB-231 cells. (A) Western blot analysis of p-PI3K, AKT, mTOR, p-mTOR^Ser2448^ in MDA-MB-231cells treated with 1.25, 2.5, 5 µM of AM879 for 24 h. The relative p-PI3K and p-mTOR^Ser2448^ expression levels were quantified by normalisation to β-actin. **p < 0.01, ***p < 0.001 and ****p < 0.0001. (B) Representative immunofluorescence images of LC3 puncta in MDA-MB-231 cells transiently expressing GFP-mRFP-LC3 plasmid followed by treatment of AM879 for 24 h. The number of LC3 puncta per cell was quantified by Image J software, ***p < 0.001. Scale bar = 10 μm. (C)Western blot analysis of ULK1, p-ULK1^Ser555^, Beclin 1, SQSTM/p62 and LC3 in MDA-MB-231cells treated with 1.25, 2.5, 5 µM of AM879 for 24 h. The relative Beclin1 and LC3-II expression levels were quantified by normalisation to β-actin. *p < 0.05, **p < 0.01 and ****p < 0.0001.

To further elucidate the antiproliferative mechanism of AM879, we conducted a literature review of ATAD2^28^ and speculated that the AKT signalling pathway might play an important role in ATAD2 inhibition-induced cell death. Interestingly, AM879 could inhibit PI3K-AKT-mTOR signalling with obviously downregulated expression of PI3K, AKT, mTOR and mTOR^Ser2448^ (Figure 6(A)). Considering that the AKT-mTOR signalling pathway is also important in regulating autophagy, we next evaluated whether AM879 can induce autophagy. We found obvious aggregation of LC3 puncta following AM879 treatment, and AM879 increased the ratio of LC3 fluorescence, indicating induction of autophagy (Figure 6(B)). Besides, AM879 resulted in the elevation of ULK1, p-ULK1, Beclin 1 and LC3-II in a dose-dependent manner, as well as degradation of SQSTM1/p62 (Figure 6(C)). These results indicated that AM879 induced autophagy via the PI3K-AKT-mTOR-ULK1 pathway.

## 4. Conclusions

Histone acetylation plays a central epigenetic role in the organisation of chromatin domains and the regulation of gene expression. ATAD2 has been reported to play an important role in the processes of numerous cancers and validated to be a promising therapeutic target. In this study, we performed a comprehensive screening strategy based on virtual screening and biochemical analyses to search for leads of ATAD2 Bromodomain inhibitor. AM879 potently inhibited ATAD2 with IC_50_ of 3,565 nM, and the MTT assay substantiated that AM879 showed potent antiproliferatory activity with IC_50_ of 2.43 µM. The selectivity assay documented that AM879 is a selective ATAD2 inhibitor. The results of molecular docking, molecular dynamics simulation and binding free energy calculation indicated AM879 is a potential substrate competitive binding inhibitor interacting with the residues Tyr1021, Asn1064 via hydrogen bonds interactions. In addition, AM879 suppressed the expression of c-Myc, and induced significant apoptosis. Additionally, AM978 could induce autophagy via PI3K-AKT-mTOR signalling in MDA-MB-231 cells. This study demonstrates an efficient Structure-Based virtual screening procedure used to successfully identify a novel selective ATAD2 inhibitor. The results may provide meaningful clues for the further development of ATAD2 inhibitors with novel scaffolds to treat breast cancer.
